# Rapid Analysis of Vessel Elements (RAVE): A Tool for Studying Physiologic, Pathologic and Tumor Angiogenesis

**DOI:** 10.1371/journal.pone.0020807

**Published:** 2011-06-09

**Authors:** Marc E. Seaman, Shayn M. Peirce, Kimberly Kelly

**Affiliations:** 1 Department of Biomedical Engineering, University of Virginia, Charlottesville, Virginia, United States of America; 2 Robert M. Berne Cardiovascular Research Center, University of Virginia, Charlottesville, Virginia, United States of America; Leiden University Medical Center, Netherlands

## Abstract

Quantification of microvascular network structure is important in a myriad of emerging research fields including microvessel remodeling in response to ischemia and drug therapy, tumor angiogenesis, and retinopathy. To mitigate analyst-specific variation in measurements and to ensure that measurements represent actual changes in vessel network structure and morphology, a reliable and automatic tool for quantifying microvascular network architecture is needed. Moreover, an analysis tool capable of acquiring and processing large data sets will facilitate advanced computational analysis and simulation of microvascular growth and remodeling processes and enable more high throughput discovery. To this end, we have produced an automatic and rapid vessel detection and quantification system using a MATLAB graphical user interface (GUI) that vastly reduces time spent on analysis and greatly increases repeatability. Analysis yields numerical measures of vessel volume fraction, vessel length density, fractal dimension (a measure of tortuosity), and radii of murine vascular networks. Because our GUI is open sourced to all, it can be easily modified to measure parameters such as percent coverage of non-endothelial cells, number of loops in a vascular bed, amount of perfusion and two-dimensional branch angle. Importantly, the GUI is compatible with standard fluorescent staining and imaging protocols, but also has utility analyzing brightfield vascular images, obtained, for example, in dorsal skinfold chambers. A manually measured image can be typically completed in 20 minutes to 1 hour. In stark comparison, using our GUI, image analysis time is reduced to around 1 minute. This drastic reduction in analysis time coupled with increased repeatability makes this tool valuable for all vessel research especially those requiring rapid and reproducible results, such as anti-angiogenic drug screening.

## Introduction

The study of vascular remodeling has long been an interest of researchers in many fields including oncology[1], ocular neovascular diseases [2]–[5], peripheral arterial disease (PAD) [6]–[8], tissue regeneration [9]–[11], and wound healing [12]–[14]. In oncology tumor vasculature is structurally and functionally aberrant, resulting in areas of hypoxia and an overall increase in interstitial fluid pressure [15]. Hypoxia and increased interstitial fluid pressure are thought to be barriers to drug delivery and effective anti-cancer therapy [15], therefore studying the role of vasculature is of vital importance. In PAD, tissue revascularization through exogenous application of angiogenic mitogens such as vascular endothelial growth factor (VEGF) and basic fibrobloast growth factor (bFGF) represents an interesting therapeutic approach [16]. In the preclinical setting research tools are needed to study the angiogenic effect of different compounds, especially those which enhance accuracy and speed of analysis.

As angiogenesis is vital to studying the progression or mitigation of disease, a quick and accurate analysis tool for in vivo models of angiogenesis is needed. Vessel analysis software is commercially available with some software packages being sold as optional add-ons to certain microscope systems such as Nikon's NIS-Elements (www.nis-elements.com) or Mauna Kei Technologies' Cellvizio (www.maunakeatech.com). However, these programs are costly and not open sourced to all, making them difficult to manipulate and adapt based on specific research needs. Some researchers have published manipulations to commercially available software [17], [18], but their efforts are not accessible unless the respective software has been purchased. An example of in vitro vessel MATLAB image processing software developed in-house is AngioQuant [19]. In the university or small research lab setting, most in vivo or ex vivo vessel analysis is completed manually with minimal computer assistance. This long, tedious analysis often takes 20 minutes to 1 hour per image and suffers from analyst measurement variation. Vessel analysis software can drastically reduce the investigator's time commitment, leading to more rapid discoveries and ultimately better patient care. A decrease in analysis time must not drastically affect the accuracy of measurement. However, incremental or slight increase in error can be tolerated, especially if analysis time is significantly minimized.

**Figure 1:**
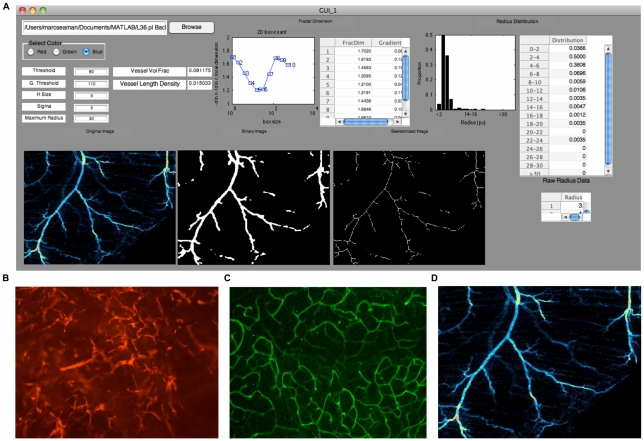
Overview of RAVE GUI. A. A representative screenshot of RAVE . The user is able to input parameters in the editable fields on the left and monitor the binarization and skeltonization of the image before ending the fitting process and recording data. The “Binary Image” is shown after being binarized, smoothed using a Gaussian filter, then binarized again. B. In vivo image of tumor associated vasculature can be analyzed by RAVE. C. Ex vivo whole mounted pancreas vasculature was analyzed by RAVE. D. Ex vivo whole mounted spinotrapezius images was analyzed.

Because MATLAB is a common engineering and science computing language accessible to most investigators, an open-source vessel analysis tool designed in MATLAB can be used and/or customized easily by all who are interested. As is, our software Rapid Analysis of Vessel Elements (RAVE), shown in Figure 1, quickly and accurately analyzes crucial vessel elements in understanding physiologic and pathologic angiogenesis in second to minutes. Using pancreatic tumor vasculature as a relevant test-bed for validating our new tool, RAVE rapidly detects a significant increase in vessel volume fraction (VVF), vessel length density (VLD), vessel radius and fractal dimension of pancreatic tumor vasculature compared to normal pancreatic vasculature.

## Results and Discussion

### Validation of Vessel Volume Fraction and Vessel Length Density calculations

As multiple fields use both ex vivo and in vivo imaging methods for analysis, we decided to determine whether RAVE could accurately and precisely quantitate vessel parameters including VVF and VLD. Therefore, we used an ex vivo assay employing immunofluroescent staining of excised, whole mounted spinotrapezius muscle tissue. For procedure details see Methods S1. The burgeoning in vivo molecular imaging field is poised to make rapid advances in understanding vessel biology, therefore, we also used an in vivo tumor model. A tumor was implanted in a dorsal skinfold chamber [20] and vessels were visualized intravitally after injection of tetramethylrhodamine labeled 2×10^6^ MW dextran. For complete methods, see Methods S1.

**Figure 2:**
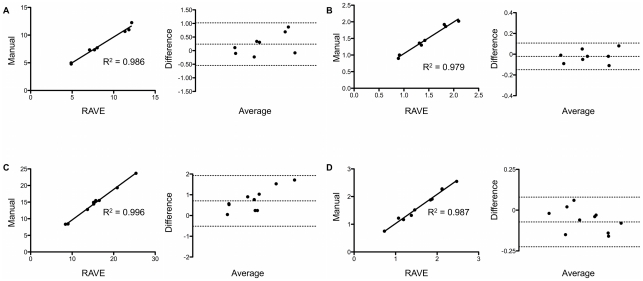
Validation of VVF and VLD. Ex vivo (A,B) and in vivo (C,D) comparison of RAVE and manually calculated VVF (A, C) and VLD (B, D). In each subpanel, the left and right plots present correlation and Bland-Altman analysis, respectively. Data is shown for multiple image fields in a single animal for both ex vivo and in vivo analyses.

Validation of manual and RAVE calculated Vessel Volume Fraction and Vessel Length Density was accomplished by plotting manually and RAVE measured VVF and VLD against each other, then calculating a correlation coefficient (Figure 2, insets). In both VVF and VLD, in vivo images had higher correlation coefficients (0.996 and 0.987, respectively) indicating that RAVE more accurately calculated VVF and VLD in intravital images of tumor microvasculature. Although slightly less, the correlation coefficient of VVF and VLD in ex vivo images of the microvasculature contained within mouse spinotrapezius muscle (0.986 and 0.979) was well above 0.95, lending confidence in the accuracy of RAVE calculated VVF and VLD compared to manual methods, both in vivo and ex vivo.

In order to recommend replacing an established method with confidence, agreement between the two methods must be achieved. Therefore, to provide a more rigorous analysis and understanding of RAVE calculated VVF and VLD, we also analyzed the same data using Bland-Altman Analysis [21]. The Bland-Altman statistical method addresses some issues that are inherent when comparing a new method to a more established or “gold standard” method. One of which is that the often-reported r-value is a measure of the strength of correlation, not a measure of agreement.

Bland-Altman plots of ex vivo (Figures 2A and 2B) and in vivo (Figures 2C and 2D) data show RAVE accurately and precisely calculated VVF and VLD. The mean difference (bias) between manually and RAVE calculated VVF in the ex vivo image set was minimal (0.2388%) and the upper and lower limits of agreement were 1.0207 and -0.5432%, respectively. Bias indicates whether the new method tends to over or under-estimate a particular measurement when compared to the standard method. In this instance, RAVE tended to over-estimate ex vivo VVF measurements by a slight amount, only 0.2388%. The upper and lower limits of agreement represent two standard deviations above or below the bias, respectively. Ex vivo VLD calculations have about an order of magnitude reduction in bias (−0.0213%). Likewise, the upper (0.1072%) and lower (−0.1497%) limits of agreement were reduced in VLD calculations.

Data acquired from intravital images were not as precise, but were similarly accurate. The bias of VVF calculations was 0.705%, while the upper and lower limits of agreement were 1.9251 and −0.5151%, respectively. The bias of the VLD calculations was small, only −0.0725%; the upper and lower limits of agreement were relatively small, 0.0797 and −0.2247%, respectively.

Bland-Altman analysis was particularly useful in showing the relative magnitude of noise when using RAVE to analyze data sets. When increases or decreases of VVF or VLD greater than approximately 2% are detected by RAVE, there is confidence that these are actual changes in VVF and/or VLD, and not simply a product of the small amount of noise introduced by RAVE. Most vascular changes are larger than this approximate threshold, making RAVE an accurate and precise VVF and VLD measurement tool.

### Validation of manual and RAVE calculated distribution of radius

Another important measure of vascular remodeling is vessel radius, usually reported as either mean vessel radius for the entire image, binned radius distributions, or changes to an individual vessel's radius (over time or prior to and following a specific treatment). In order to demonstrate that RAVE could be used to analyze this metric of vascular remodeling, we sought to compare RAVE to manually measured vessel distributions. The distribution of radii allows the investigator to assess general trends in the image area throughout the microvascular network.

**Figure 3:**
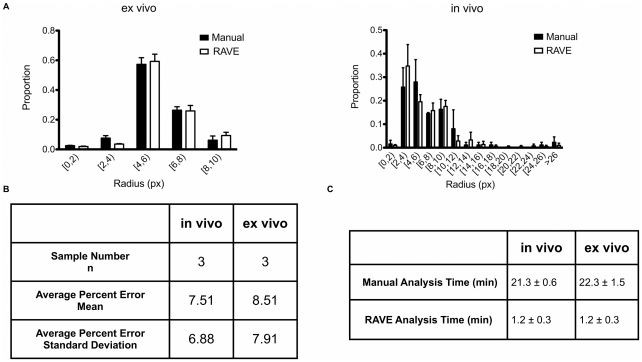
Comparison of RAVE and manually calculated radii. A, left. An ex vivo image set (n = 3 fields, single animal) comparison of manual and RAVE calculated radius distributions. A, right. An in vivo image set (n = 3 fields, single animal) comparison of manual and RAVE calculated radius distributions. B. Analysis of RAVE and manually calculated radii are compared for both in vivo and ex vivo images using mean and standard deviation. A sample size (n) of 3 (single animal, different fields) was used to calculate percent error of the means and standard deviations. C. Average analysis time for manual and RAVE calculated radius distributions. Standard deviations are listed after the “±” symbol. Manual analysis time included drawing the grid, measuring the radii and binning in using Excel software. RAVE analysis time included loading the image and pasting the data to Excel, so that comparisons between the two methods could be made.

Ex vivo images were analyzed by overlaying a grid composed of 3000 (ex vivo) or 4000 (in vivo) px^2^ boxes on the images to be analyzed. For details, see Methods S1. Comparisons between RAVE and manually obtained radius distributions were made using the mean and standard deviation of the assumed normally distributed data. Qualitatively, the RAVE-attained radius distribution matched well with the manually measured radius distribution. A histogram of radius distributions for an ex vivo and in vivo image set is shown in Figure 3A. The relative distributions coordinated across a wide range of pixel radius bins, where maximas and minimas generally occurred in the same bins for ex vivo and in vivo images. The mean and standard deviation comparison results were summarized in Figure 3B. Percent error of distribution means and standard deviations were quite small (less than 10.0%), as the largest percent error was 8.51. In vivo mean and standard deviation percent errors were 7.51 and 6.88, respectively. Compared to ex vivo percent errors (8.51 and 7.91, respectively), in vivo radius distribution determination was slightly more accurate. This is in accordance with previous comparative observations between in vivo and ex vivo images. RAVE was slightly less accurate determining the radius distribution of ex vivo images; however, the percent errors were relatively small, not surpassing 10.0%.

The analysis time saving capacity of RAVE is best demonstrated when determining the radius distribution parameter. This process of measuring and binning radii is quite laborious when completed manually, but these steps are integrated by RAVE, resulting in a much reduced analysis time (Figure 3C). Manually calculated data took about 20 minutes to complete. For each type of image (in or ex vivo), three representative images were analyzed, bringing the total time spent to roughly two hours. RAVE calculated radius distributions took just over one minute, so total analysis would take about six minutes. RAVE is an accurate and especially time conserving method for qualitatively and quantitatively observing shifts in vessel radius.

### RAVE calculated Fractal Dimension

Fractal dimension represents another metric that is used to describe vascular remodeling [18], [22], [23]. RAVE implements the widely-used box counting method [24]. Details of this implementation are discussed in the “Design Implementation” section. When a completely white image was analyzed, RAVE calculated the appropriate fractal dimension for a 2D shape, 2. Likewise, when a 1-pixel width line was analyzed, RAVE returned a fractal dimension of 1. RAVE reproducibly calculated the fractal dimension of several tests shapes and therefore is useful in determining vascular fractal dimension.

### Detecting changes in angiogenic vessel architecture using RAVE

**Figure 4:**
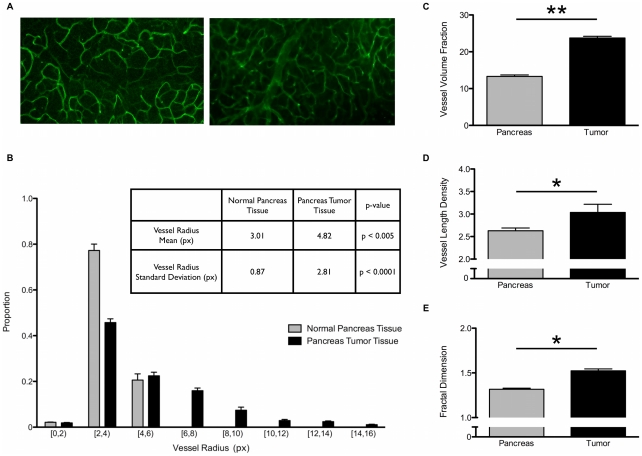
Detecting vascular architectural shifts in VVF, VLD, fractal dimension and radius. A, left. Representative normal pancreas vasculature imaged used in this analysis. A, right. Representative pancreatic tumor vasculature image used in this analysis. B. The shift in size of vessels is captured and mean and standard deviation data is shown on the inset table. C. Vessel Volume Fraction is displayed. D. Vessel Length Density is displayed. E. Fractal dimension is displayed. ** p-value <0.005, * p-value <0.05. p-values calculated by paired, one-tail student's t-test. Three images from one animal were used in each group (normal pancreas and tumor).

The hallmarks of tumor angiogenesis include increased VVF, VLD, fractal dimension and vessel radius. To demonstrate the utility of RAVE, we sought to determine if RAVE could rapidly detect changes in pancreatic tumor vasculature, which qualitatively appear unchanged. The lack of obvious vessel morphology changes can be seen by studying the representative images in Figure 4A. However, RAVE can distinguish normal pancreatic vasculature from tumor-associated angiogenic vasculature. Characteristic increases in VVF, VLD, vessel radius, and tortuosity were captured by RAVE. In one such example summarized in Figure 4, whole mounted normal pancreas (Figure 4A, left) and tumor vasculature (Figure 4A, right) were analyzed for shifts in vessel architectural parameters. RAVE was able to significantly distinguish between normal and tumor-associated vasculature. VVF nearly doubled in tumor vasculature, significantly increasing from 13.3 to 28.8% (Figure 4C). Increases in VLD and fractal dimension were not as drastic, but their effects were still easily measured and observed (Figures 4D and 4E). VLD significantly increased from 2.63 to 3.04 and fractal dimension significantly increased from 1.32 to 1.52. The fractal dimension calculations recapitulate what has previously been published in the literature. In two examples of oral and colon cancer, control microvasculature had a fractal dimension around 1.1 and tumor microvasculature ranged in fractal dimension from 1.7 to 1.9 [22], [25]. Discrepancies arise because pancreatic tumors are hypovascular compared to most other tumors' microvasculature. In a pancreatic cancer example, live tumors were imaged by exteriorizing the pancreas and associated tumor, then visualizing vasculature using a fluorescent blood pool agent, AngioSense 680. The fractal dimension of normal pancreas microvasculature was 1.36 and pancreatic tumor vasculature was 1.43 [18]. This matches very well with our independently measured shift in fractal dimension from 1.32 to 1.52. In addition to changes in VVF, VLD, and fractal dimension, a corresponding shift in vessel radius was captured by RAVE (Figure 4B). Both proportional peaks occurred in the [2,4) pixel bin. However, after inspecting the distributions, one sees that a higher proportion of normal pancreas vessels occupy smaller bins (0 to 4 pixels), while a greater proportion of pancreatic tumor vessels occupy larger bins (4 to 16 pixels). The trends shown in Figure 4B correlate well with previously published shifts in pancreatic tumor microvessel radius distribution [18]. There is a significant change in normal and tumor distributions, measured by mean vessel radius. As expected, pancreatic tumor vessels have 1.6-fold greater mean vessel radius and 3.2-fold greater standard deviation when compared to normal pancreatic vessels. RAVE can be successfully used to rapidly and quickly monitor modulation of vessel architecture whether as a result of accelerating tumor angiogenesis or vessel normalization induced by anti-angiogenic compounds. The ability to demarcate tumor vasculature, which is not immediately obvious, coupled with its rapid and accurate analysis capabilities make RAVE an excellent tool for investigators who study vascular remodeling.

### Customization and other applications

As such, RAVE is designed to analyze two-dimensional images. Parameters such as VVF, VLD, fractal dimension and radius distribution are important in many fields, but three-dimensional analysis is sometimes needed. Three-dimensional analysis is especially needed when analyzing flow and other rheological measurements. A rheologically important parameter is 3D branch angle, accounting for vessels tendencies to move into and out of a particular z-plane. Using sufficiently thin vibratome sections or tissues such as the murine spinotrapezius can reduce information lost in the third dimension. Although RAVE cannot be easily modified to accomplish 3D mapping as used in expensive systems like Neurolucida [26], one could still modify RAVE to calculate 2D branch angle. A modification to calculate branch angle might include user prescribed bifurcations, followed by execution of an algorithm similar to the vessel radius algorithm described here. Vascular network structure resulting from branching can be described by generational or Strahler ordering. RAVE does not currently determine network structure, but could be modified to display results on its GUI. Other parameters that can be assessed with simple modifications to RAVE include percent coverage of a particular immunofluorescently labeled cell type, number of vessel loops and amount of perfusion.

## Methods

### Ethics Statement

All animal experiments were approved by the University of Virginia Institutional Animal Care and Use Committee. Work was completed on Protocols 3731 and 3459.

### GUI construction

The RAVE GUI was created with MATLAB's “guide” function. Information on how to download RAVE, its manual and a test data set is contained in Supporting Information S1. The RAVE GUI directly outputs vessel volume fraction, vessel length density and radius distribution. Fractal dimension can be determined by looking at the plot and table in the Fractal Dimension section. Figure 1A provides a screenshot of the RAVE interface.

The file to be analyzed is selected by clicking on the “Browse” button. Once the image is loaded, it will appear on all three image panes (Original Image, Binary Image, and Skeletonized Image). These image panes can be used to visually inspect how well the thresholded binary and skeletonized images represent the Original Image. The Threshold, Gaussian Threshold (G. Threshold), H Size, Sigma and Maximum Radius parameters can be adjusted. Their meaning and considerations will be discussed further in this section. Each time a parameter is adjusted the display and calculations are updated. The Fractal Dimension section contains a plot and table that allows the user to determine the correct fractal dimension. Guidelines for determining fractal dimension will be discussed in the following sections. The Radius Distribution section displays the radius data in table and histogram form. Each bar represents a range of 2 pixels, with the exception being the “>30” bar. Also, the bounds of the bins are (x,y], where x is the first number in the range and y is the last. For instance, a measured radius of 4 pixels would be placed in the “2–4 pixel” bin.

RAVE is capable of analyzing a variety of images gathered from numerous modalities; however, one constraint, for optimal calculation speed, is that image size should be less than 600 pixels in either or both dimensions. Of course, larger images can be analyzed, but their processing time can be quite cumbersome when parameters are being adjusted. One strategy to avoid this delay in processing time is to adjust parameters using a scaled down image, then once parameters are set, using the full sized image. A sample of images that can be analyzed with RAVE is shown in Figure 1B–D. Each of these sample images was used in the validation of RAVE. Figure 1B is an in vivo image taken using an intravital microscope. A vibratome section of the pancreas is shown in Figure 1C. Vibratome sections were stained with an anti-CD31 antibody and imaged with a confocal microscope. In a similar manner, the extremely thin spinotrapezius muscle is stained with alpha-smooth muscle actin antibody, whole mounted and imaged with a confocal microscope. Additionally, brightfield images of vasculature can be analyzed with a few minor pre-processing steps, which are outlined in the Manual available online.

### Vessel Volume Fraction Calculation

VVF represents the fraction of each image space that contains vessels. To calculate VVF, the image to be analyzed is binarized using the value input in the Threshold space. In short, MATLAB moves pixel-by-pixel evaluating if the current pixel is above the threshold. A binary image is produced; if the pixel is above the threshold, it will be turned white and if it is below, black. This binarized image is quite jagged and leads to problems with skeletonization. Thus, the image must be smoothed using additional filtering techniques. An unsmoothed and smoothed binary image is shown in the manual. Gaussian Threshold (G. Threshold), H Size and Sigma are used to create the smoothed binarized image. The raw binarized image is passed through a rotationally symmetric low-pass filter of size “H size” and standard deviation “Sigma” using the “Gaussian” type fspecial MATLAB function. The output of this Gaussian filter is not a binarized image, instead it must be binarized again using the threshold specified in the “G. Threshold” space. The resulting final binarized image is a smooth representation of the original image. After the smoothed binarized image is obtained, the total number of white pixels in the image is summed. This sum is divided by the total number of pixels in the image and VVF is calculated.

### Vessel Length Density Calculation

VLD represents the total length of all vessels divided by total pixel area. To calculate VLD, the smoothed binary image from above was skeletonized using the ‘skel’ type ‘bwmorph’ MATLAB function. In skeletonization, all continuous white shapes are reduced to a 1 pixel-wide segments. The result is a collection of lines representing the midlines of all vessel shapes. Since this line is 1 pixel wide, the sum represents the total length of vessels. The sum of white pixels is divided by the total number of pixels to produce the VLD calculation.

### Vessel Radius Calculation

**Figure 5:**
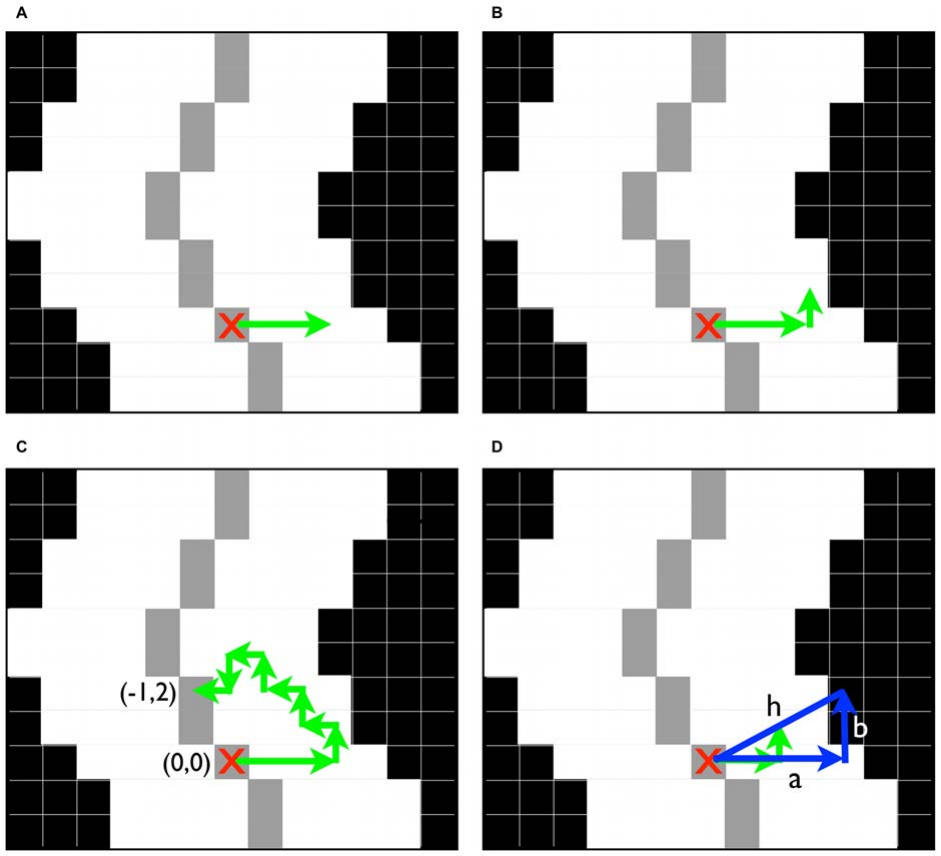
Schematic for the determination of vessel radius algorithm. A. The starting or current position (0,0) is shown by the red “X”. First, the sampling moves three pixels to the right (3,0). If a value of 105 is not found, the algorithm begins its counter-clockwise search for a pixel of value 105. B. Counter-clockwise movement is initiated by moving to pixel (3,1) for sampling. C. Counter-clockwise movements until a 105-valued pixel is found at position (−1,2). D. Walking in an orthogonally prescribed direction is completed until a black (0-valued) pixel is found. The green arrows represent the first “unsuccessful” walk and blue arrows represent the second “successful” walk. Once a black pixel is found, walking ceases and radius is calculated from the hypotenuse (h) of Pythagorean Theorem, where “a” and “b” represent the horizontal and vertical walking distance, respectively.

To calculate the radius, the smoothed binary and skeletonized images were used to create a composite image. A composite image is obtained as a means to distinguish the center line (gray), and the vessel (white) from the background of the image (black). From this, one can determine vessel trajectory and the edge of the vessel. First, the skeleonized white (255) pixels were changed to pixels with values of 150. The 150-value skeletonized image was subtracted from the smoothed binary image, creating a composite image with three color values, black (0), grey (105) and white (255). The resulting composite image is shown in Figure 5A.

Next, the radius at each grey (105) pixel was determined. A schematic depiction of radius determination is shown in Figure 5A–D. To determine the local angle of the vessel, an algorithm was applied to each pixel. The currently interrogated pixel has coordinates (0,0) and is symbolized in Figure 5 with a red “X”. First, a location three pixels to the right (3,0) was sampled for color value. If the color value is 105, then the angle is determined to be 0°, since the local angle of the gray midline would be horizontal or 0°. If the value is anything other than 105, then the algorithm moves in a counter-clockwise fashion and the value of pixel (3,1) is sampled (Figure 5B). If it is not 105, then the next position to be sampled is (2,1) and this counter-clockwise movement is continued until a pixel valued at 105 is located (Figure 5C). Once a 105-valued pixel is found its coordinates are used to determine in which direction the radius will be calculated. The correct radius is calculated orthogonal to the direction of the midline of the vessel. Using directional cues from the above search, an orthogonal walk is executed until the first black (0) pixel is identified. In the example depicted in Figure 5, if the 105-valued pixel is located at position (−1,2) as in Figure 5C, then the radius will be determined through walks of 2 pixels right (+2) and 1 pixel up (+1) relative to position (0,0). Radius walking is shown in Figure 5D. The first walk does not land on a black pixel (via green arrows), so the search continues (blue arrows), eventually finding a black pixel at +4 pixels right and +2 pixels up. Once a black pixel is encountered, walking ceases and the radius is calculated by solving for the hypotenuse (h) in Pythagorean theorem, where legs a and b represent the distance walked in the horizontal and vertical directions, respectively. As depicted in Figure 5D, the radius estimation is often fractions of a pixel greater than the true radius. These errors are inherent to pixelation of the vessel, but do not seem to affect the accuracy of radius calculation as demonstrated in the “Results” section.

Each calculated radius is concatenated onto a growing array of radii. The radius array is voided of radii above the maximum as determined by the “Maximum Radius” input. Additionally, each radius is placed into 2-pixel wide bins. The total number of counts in each bin is divided by the length of the modified radius array, giving rise to the proportion of radii contained in each bin. The binned proportion data is displayed in tabular and graphical (histogram) form on RAVE's interface.

### Fractal Dimension Calculation

Fractal dimension is calculated using the skeletonized image and the boxcount function produced by F. Moisy and published on MATLAB Central on November 21, 2006. Determining fractal dimension by box counting involves counting the total number of boxes on a grid that contain a line segment in them for grids comprised of progressively smaller boxes. The number of counts (n) versus box size (r) is plotted on a double-logarithmic plot (not shown on RAVE). The slope of that plot represents the fractal dimension. However, there are often many local and varying slopes and determining the true fractal dimension can be difficult. The true fractal dimension is located in a region of constant slope. To identify regions of constant slope, it is useful to plot the local slope (-d ln(n) / d ln (r)) as a function of box size (r). Taking the gradient of the local slope data gives an approximation of constancy. Areas of constant local slope have the smallest gradient and thus represent the true fractal dimension. In the analyzed image represented by the GUI screenshot in Figure 1A, the fractal dimension is located where the line “bottoms out” and the gradient is minimized. The fractal dimension displayed by RAVE in the Fractal Dimension output represents the slope where the gradient is minimized. Sometimes, this is not the true fractal dimension as the tails (beginning and end) of the curve usually have small gradients. The real fractal dimension lies somewhere in the middle of the plot and might not be correctly determined in the case of minimum gradients apparently located at either tail of the curve. As such, the table of fractal dimensions and their corresponding gradients will allow the user to select the correct fractal dimension. To select the correct fractal dimension locate the smallest gradient on the interior of the curve.

## Supporting Information

Methods S1 **This supplement includes image acquisition and analysis methods.**
(DOC)

Supporting Information S1 **This supplement directs users to a link where one can find the GUI, source code, user manual, and a test data set.**
(DOC)
