# Supplementary material for: Rapid Analysis of Vessel Elements (RAVE): A Tool for Studying Physiologic, Pathologic and Tumor Angiogenesis
Source: PLoS One. 2011 Jun 9;6(6):e20807. doi: 10.1371/journal.pone.0020807 (PMC3111429; doi:10.1371/journal.pone.0020807)
Supplement: Methods S1 — This supplement includes image acquisition and analysis methods. (DOC) [file pone.0020807.s001.doc]

**Supplemental Materials and Methods S1**

***Immunohistochemistry, Microscopy, and Image Generation***

All animal experiments were approved by the University of Virginia Institutional Animal Care and Use Committee.

*Tumor associated vessels*

L3.6pl pancreatic tumor cells (1.5x10^5^) were injected subcutaneously in athymic nude mice. Approximately five days after tumor cell injection, a dorsal skinfold window chamber (described in [1] with tumor implantation modifications) was installed over the developing tumor. Briefly, the animal was anesthetized  (intraperitoneal injection of ketamine/xylazine/atropine; 60/4/0.2 mg/kg), shaved and depilated, then the skin manually lifted to create a skinfold. One half of the window frame was attached to the skinfold, the top layer of skin corresponding to the window was removed, thereby exposing the cutaneous microcirculation, and finally the second half of the frame is attached and a coverslip is placed over the exposed area. One day after installation of the window chamber, vessels were visualized with tetramethylrhodamine labeled 2.0x10^6^ molecular weight dextran (Invitrogen, Carlsbad, California, USA) 5 minutes after i.v. (tailvein) injection. Images were obtained on an Eclipse 80i intravital microscope (Nikon, Melville, New York, USA; 10x).

*Pancreatic and orthotopic pancreatic tumor associated vessels*

The orthotopic pancreas tumor injection protocol was modified slightly from previously published methods [2]. Briefly, 1x10^6^ L36.pl pancreatic tumor cells were suspended in 50 μL of HBSS. Mice were anesthetized with atropine (.04-0.1 mg/kg IP) and ketamine/xylazine (60-80/4-10 mg/kg IP), then placed so that the left side-wall is closest to the surgeon. A small incision is made in the skin, then the underlying peritoneal and the pancreas is briefly externalized. The pancreas is injected with 50 μL of cell suspension, then the pancreas is placed back inside the mouse and the peritoneal cavity and outer skin are sutured. A 1-5 mm tumor develops within 10-14 days. After the tumor was palpable to the investigator, mice were euthanized and pancreata were removed and fixed in 4% paraformaldehyde for 30 minutes. Pancreata were then embedded in Tissue-Tek molds (Sakura Finetek, Torrance, California, USA) with 5% low-melt agarose. Next, thin (200μm) sections were cut using a vibratome. Sections were then stained with anti-mouse CD31 (BD Biosciences - Cat. 550274, San Jose, California, USA). After staining, sections were mounted and visualized using (Nikon need model) scanning laser confocal microscope (20x objective).

*Spinotrapezius vessels*

Male C57Bl/6 mice (Harlan Laboratories, Indianapolis, IN) were anesthetized (intraperitoneal injection of ketamine/xylazine/atropine; 60/4/0.2 mg/kg) and the left and right spinotrapezius muscles were harvested as previously described [3]. Briefly, a horse-shoe shaped incision was made through the dorsal skin 5mm caudal to the left shoulder blade. Blunt dissection was used to separate the white fat-pad from the underlying spinotrapezius muscle to expose the muscle. The muscle was stripped of fascia, undermined and surgically excised. Harvested muscles were washed in PBS + 0.1% saponin (3 x 15 minutes), and then stained overnight at 4°C with 1A4 antibodies conjugated to FITC (Sigma; diluted 1:500 in antibody solution) to identify vessels. After immunostaining, tissues were washed in PBS + 0.1% saponin (3 x 15 minutes) then whole-mounted on gelatin-coated slides. Digital images of immunolabeled spinotrapezius tissues were acquired using confocal microscopy (Nikon, TE200-E2; 10x objective). For ease of visualization, Nikon C1 software was used to display image intensity according to a color map, where white and red represent the highest fluorescent intensities and blue/black represent the lowest intensities.

***Bland-Altman Analysis***

Bland-Altman plots were constructed following the methods set forth by Martin Bland and Douglas Altman [4]. For each image, the average of the two measurements (manual and RAVE calculated) was assigned as the x-axis variable and the difference between the two measurements was plotted is the y-axis variable. Coordinates are as follows:


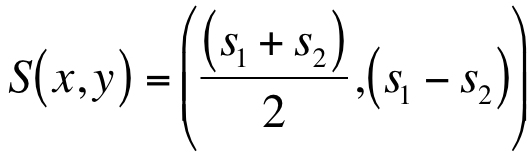


The bias (
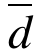
) represents the average difference between the two measurements.


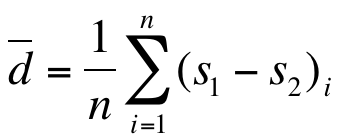


The upper and lower limits of agreement are calculated as follows:


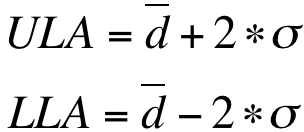


where:


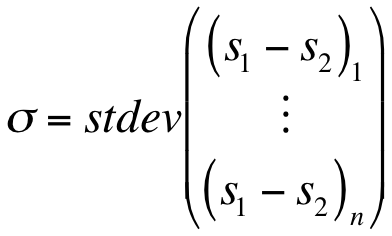


***Radius Distribution Validation Analysis***

Three ex vivo and three in vivo images were overlaid with a grid of squares of 3000 and 4000 px^2^, respectively. The vessel radius was manually measured at each vessel-grid intersection (random sampling). The distributions of manually calculated radii were compared to the distributions calculated by RAVE. Assuming the radius distributions are normally distributed, the mean and standard deviation characterize the distribution. Distribution mean and standard deviation were used to compare and discuss accuracy between the two methods.

1. Leunig M, Yuan F, Menger MD, Boucher Y, Goetz AE et al. (1992) Angiogenesis, microvascular architecture, microhemodynamics, and interstitial fluid pressure during early growth of human adenocarcinoma LS174T in SCID mice. Cancer Res 52: 6553-6560.

2. Kim MP, Evans DB, Wang H, Abbruzzese JL, Fleming JB et al. (2009) Generation of orthotopic and heterotopic human pancreatic cancer xenografts in immunodeficient mice. Nat Protoc 4: 1670-1680.

3. Bailey AM, Thorne BC, Peirce SM (2007) Multi-cell agent-based simulation of the microvasculature to study the dynamics of circulating inflammatory cell trafficking. Ann Biomed Eng 35: 916-936.

4. Bland JM, Altman DG (1986) Statistical methods for assessing agreement between two methods of clinical measurement. Lancet 1: 307-310.
