# Supplementary material for: Rapid Analysis of Vessel Elements (RAVE): A Tool for Studying Physiologic, Pathologic and Tumor Angiogenesis
Source: PLoS One. 2011 Jun 9;6(6):e20807. doi: 10.1371/journal.pone.0020807 (PMC3111429; doi:10.1371/journal.pone.0020807)
Supplement: Supporting Information S1 — This supplement directs users to a link where one can find the GUI, source code, user manual, and a test data set. (DOC) [file pone.0020807.s002.doc]

To access the GUI, source code, user manual and training data set go to

http://people.virginia.edu/~mes4uf/RAVE.html
